# Medical College Convention

**Published:** 1870-01

**Authors:** 


### Editorial Department.

### Medical College Convention.

To the Trustees and Faculties of the Medical Colleges in the United States:

The undersigned Committee, in accordance with the instructions of the Convention of Delegates from the M dical Colleges, held in Cincinnati in May, 1866, respectfully and earnestly invite you to send Delegates to a Convention to be held in Washington on the Friday preceding the first Tuesday in May, 1870, for the purpose of considering all subjects connected with medical college education, and procuring the co-operation of schools in carrying out a uniform system of medical instruction. It is very desirable that every regular medical college in the country should be represented in this Convention.

Chicago, Ill., Dec. 22, 1869.

N. S. DAVIS,

S. D. GROSS

GEO C. BLACKMAN,

F. DONALDSON,

*Committee.*

